# Extended Blahut–Arimoto Algorithm for Semantic Rate-Distortion Function

**DOI:** 10.3390/e27060651

**Published:** 2025-06-18

**Authors:** Yuxin Han, Yang Liu, Yaping Sun, Kai Niu, Nan Ma, Shuguang Cui, Ping Zhang

**Affiliations:** 1 Key Laboratory of Universal Wireless Communications, Ministry of Education, Beijing University of Posts and Telecommunications, Beijing 100876, China; hanyx@bupt.edu.cn (Y.H.); liuyang@bupt.edu.cn (Y.L.); 2 Department of Broadband Communication, Pengcheng Laboratory, Shenzhen 518055, China; sunyp@pcl.ac.cn (Y.S.); 3 Key Laboratory of Networking and Switching Technology, Beijing University of Posts and Telecommunications, Beijing 100876, China; manan@bupt.edu.cn (N.M.); pzhang@bupt.edu.cn (P.Z.); 4 School of Science and Engineering (SSE) and the Future Network of Intelligent Institute (FNii), The Chinese University of Hong Kong (Shenzhen), Shenzhen 518172, China; shuguangcui@cuhk.edu.cn (S.C.)

**Keywords:** Blahut–Arimoto algorithm, semantic rate-distortion function, semantic information theory, semantic knowledge base

## Abstract

Semantic communication has recently gained significant attention in theoretical analysis due to its potential to improve communication efficiency by focusing on meaning rather than exact signal reconstruction. In this paper, we extend the Blahut–Arimoto (BA) algorithm, a fundamental method in classical information theory (CIT) for computing the rate-distortion (RD) function, to semantic communication by proposing the extended Blahut–Arimoto (EBA) algorithm, which iteratively updates transition and reconstruction distributions to calculate the semantic RD function based on synonymous mapping in semantic information theory (SIT). To address scenarios where synonymous mappings are unknown, we develop an optimization framework that combines the EBA algorithm with simulated annealing. Initialized with a syntactic mapping, the framework progressively merges syntactic symbols and identifies the mapping with a maximum synonymous number that satisfies objective constraints. Furthermore, by considering the semantic knowledge base (SKB) as a specific instance of synonymous mapping, the EBA algorithm provides a theoretical approach for analyzing and predicting the SKB size. Numerical results validate the effectiveness of the EBA algorithm. For Gaussian sources, the semantic RD function decreases with an increasing synonymous number and becomes significantly lower than its classical counterpart. Additionally, analysis on the CUB dataset demonstrates that larger SKB sizes lead to higher semantic communication compression efficiency.

## 1. Introduction

Recent years have witnessed the rapid development of semantic communication, which has emerged as a promising paradigm for the next-generation communication system [1]. Unlike the traditional communication system that focuses on transmitting symbols, semantic communication aims to recover the message that matches the meaning of the transmitted information [2]. This paradigm enables more efficient data transmission by focusing on transporting and delivering the meaning of messages, potentially reducing communication overhead while maintaining the essential meaning of the information [3,4,5,6].

Classical information theory (CIT), established by Shannon in 1948, has served as the cornerstone for modern digital communication [7]. In CIT, the rate-distortion (RD) theory characterizes the fundamental trade-off between the rate and the distortion in lossy compression systems [8,9,10]. The Blahut–Arimoto (BA) algorithm has been established as a prevailing numerical method in CIT, which is capable of computing the RD function for arbitrary discrete source models [11,12]. Variants and acceleration techniques of the BA algorithm have been developed [13,14,15,16,17], and a mapping approach has been introduced for continuous sources [18].

Recent research suggests that the RD-perception function plays an important role in semantic communication [19,20,21,22,23]. More recently, Niu and Zhang extended CIT to semantic communication, referred to as the semantic information theory (SIT) [24]. By introducing the synonymous mapping, information measures (such as semantic entropy, which is consistent with that used in large language models [25]) and coding theorems for the semantic communication system were established, thus providing a mathematical foundation for guiding the design of the semantic communication system. In their work, the analytical expression of the semantic RD function for Gaussian sources is derived; however, computing the semantic RD function for other source models remains challenging, which motivates us to extend the classical BA algorithm to the semantic communication system.

In this paper, we propose an extension of the BA algorithm based on the SIT, referred to as the extended Blahut–Arimoto (EBA) algorithm, such that the semantic RD function can be directly calculated with any given source model. The main contributions of this paper are summarized as follows.

- First, based on the synonymous mapping in SIT, the EBA algorithm is proposed by extending the classical BA algorithm to the semantic communication system. Similar to the BA algorithm, the EBA algorithm is also an iterative procedure that converges to the semantic RD function through alternating optimization of the transition and reconstruction distributions. The convergence is guaranteed by the convexity property of the semantic RD function, providing an efficient method for computing the semantic RD function for arbitrary discrete source models.
- Then, starting from a syntactic mapping, an optimization framework is developed for scenarios with unknown synonymous mappings. The framework combines the EBA algorithm with simulated annealing [26] to progressively merge syntactic symbols and identify the mapping with maximum synonymous number that satisfies objective constraints, enabling the discovery of optimal semantic representations that balance compression efficiency and distortion.
- Furthermore, by considering the semantic knowledge base (SKB) as a specific instance of synonymous mapping, the EBA algorithm provides a theoretical approach for analyzing and predicting the SKB size. Using the CUB dataset [27], experimental results indicate that increasing the SKB size directly improves semantic communication compression efficiency, thereby validating the critical role of SKB in enhancing transmission performance.

The remainder of this paper is organized as follows. Section 2 reviews the semantic RD function in SIT and introduces the classical BA algorithm. Section 3 presents the EBA algorithm and details its optimization framework for finding the synonymous mapping with maximum synonymous number. Section 4 describes the application of the EBA to SKB. Simulation results are provided in Section 5, followed by conclusions in Section 6.

## 2. Preliminaries

### 2.1. Notation and Conventions

Throughout this paper, calligraphic letters, such as X and Y, denote sets, while lowercase letters denote elements in these sets. For a syntactic symbol, its reconstruction is denoted by $\hat{\cdot }$, and its associated semantic symbol by $\tilde{\cdot }$. The cardinality of set X is defined as |X|. Let f:X→Y denote a mapping from set X to set Y.

### 2.2. Semantic RD Function

**Figure 1:**
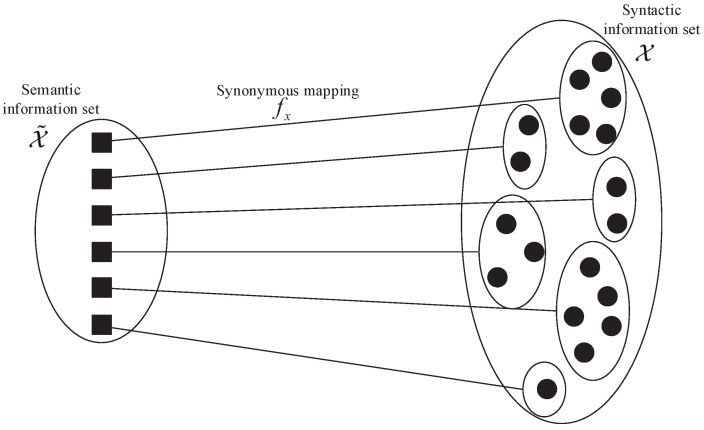
An example of synonymous mapping between semantic and syntactic information sets.

To facilitate understanding, a brief overview of the related concepts is presented first; detailed definitions can be found in [24]. Consider a syntactic information set $X=\{x_{1},\cdots ,x_{N}\}$ and its corresponding semantic information set $\tilde{X}=\left\{{\tilde{x}}_{1},\cdots ,{\tilde{x}}_{\tilde{N}}\right\}$, where $\tilde{N}\leq N$. The synonymous mapping $f_{x}:\tilde{X}\to X$ is a one-to-many mapping that partitions X into disjoint synonymous sets $\left\{X_{i_{s}}\right\}$, $i_{s}=1,2,\cdots ,\tilde{N}$, where each $X_{i_{s}}$ contains syntactic symbols sharing the same semantic meaning and $|X_{i_{s}}|=N_{i_{s}}$. As illustrated in Figure 1, each semantic symbol corresponds to an equivalent set of syntactic symbols, and there is no overlap between any two sets.

Based on the synonymous mapping, the semantic RD function is obtained by minimizing the semantic mutual information between the source and the reconstruction subject to an average semantic distortion constraint. Assume that given an i.i.d. source X∈X with its associated semantic source $\tilde{X}$, let $x_{i_{s},l}$ denote the *l*-th syntactic symbol in the $i_{s}$-th synonymous set. The corresponding source probability distributions are expressed as $p_{i_{s}l}=p\left(x_{i_{s},l}\right)$ and $p_{i_{s}}=p\left({\tilde{x}}_{i_{s}}\right)=p\left(X_{i_{s}}\right)=\sum_{l=1}^{N_{i_{s}}}p\left(x_{i_{s},l}\right)$. Let ${\hat{x}}_{j_{s},m}$ denote the *m*-th reconstruction syntactic symbol in the $j_{s}$-th synonymous set and $f_{\hat{x}}$ denote the synonymous mapping $f_{\hat{x}}:\tilde{\hat{X}}\to \hat{X}$, which is defined analogously to $f_{x}$. The test channel is characterized by transition probability $P_{j_{s}m,i_{s}l}=p\left({\hat{x}}_{j_{s},m}|x_{i_{s},l}\right)$ with semantic distortion ${\tilde{d}}_{i_{s}j_{s}}={\tilde{d}}_{s}\left({\tilde{x}}_{i_{s}},{\tilde{\hat{x}}}_{j_{s}}\right)={\tilde{d}}_{s}\left(X_{i_{s}},{\hat{X}}_{j_{s}}\right)$. For the reconstruction, the probability distributions are defined as $q_{j_{s}m}=p\left({\hat{x}}_{j_{s},m}\right)=\sum_{i_{s}=1}^{\tilde{N}}\sum_{l}p_{i_{s}l}P_{j_{s}m,i_{s}l}$ and $q_{j_{s}}=p\left({\tilde{\hat{x}}}_{j_{s}}\right)=p\left({\hat{X}}_{j_{s}}\right)=\sum_{m=1}^{N_{j_{s}}}\sum_{i_{s}}\sum_{l}p_{i_{s}l}P_{j_{s}m,i_{s}l}$. Then, the semantic RD function is defined as follows, and this problem is convex as shown in [24]:

(1) $$R_{s}\left(D\right)=\min_{f_{x},f_{\hat{x}}}\min_{P_{D}}\sum_{i_{s}}\sum_{j_{s}}\sum_{l}\sum_{m}p_{i_{s}l}P_{j_{s}m,i_{s}l}\log\frac{p_{i_{s}l}P_{j_{s}m,i_{s}l}}{\sum_{l^{\prime }}\sum_{m^{\prime }}p_{i_{s}l^{\prime }}q_{j_{s}m^{\prime }}}$$

where the test channel set $P_{D}$ is defined as

(2) $$P_{D}=\left\{P_{j_{s}m,i_{s}l}:\sum_{i_{s}}\sum_{j_{s}}\sum_{l}\sum_{m}p_{i_{s}l}P_{j_{s}m,i_{s}l}{\tilde{d}}_{i_{s}j_{s}}\leq D\right\}.$$

### 2.3. BA Algorithm

The BA algorithm is an iterative procedure for computing the RD function. Let p(x), $p(\hat{x})$, and $p(\hat{x}|x)$ denote the source distribution, reconstruction distribution, and test channel transition probability, respectively. For each fixed Lagrange multiplier $\lambda \in R^{+}$, the algorithm minimizes the RD Lagrangian $L_{\lambda }=I\left(X;\hat{X}\right)+\lambda E\left[d\left(x,\hat{x}\right)\right]$, where $I(X;\hat{X})$ represents the mutual information and $d(x,\hat{x})$ represents the distortion measure.

Starting with an initial reconstruction distribution $p(\hat{x})$, each iteration alternates between two steps: first, computing the test channel transition probability

(3) $$p\left(\hat{x}|x\right)=\frac{p\left(\hat{x}\right)e^{-\lambda d(x,\hat{x})}}{\sum_{\hat{x}}p\left(\hat{x}\right)e^{-\lambda d(x,\hat{x})}}$$

and then updating the reconstruction distribution according to

(4) $$p\left(\hat{x}\right)=\sum_{x}p\left(x\right)p\left(\hat{x}|x\right).$$

This iterative process continues until the change in the reconstruction distribution between consecutive iterations is less than a predetermined threshold. Geometrically, λ represents the slope of the tangent line of the RD curve, and each optimal solution corresponds to a point on the RD curve. By varying λ, the entire RD curve can be swept out. The convergence of this algorithm has been proved by Blahut.

## 3. Extended BA Algorithm

In this section, the EBA algorithm is presented as a systematic approach for computing the semantic RD function. We first establish the theoretical foundations of the EBA algorithm, followed by an optimization framework for finding the synonymous mapping with maximum synonymous number.

### 3.1. EBA Algorithm for the Semantic RD Function

Without loss of generality, we consider the case where the synonymous mappings $f_{x}$ and $f_{\hat{x}}$ are identical. Given a synonymous mapping that partitions the syntactic symbols into disjoint synonymous sets, the semantic RD function can be formulated as the following optimization problem:

(5) $$\min_{P_{D}}\sum_{i_{s}}\sum_{j_{s}}\sum_{l}\sum_{m}p_{i_{s}l}P_{j_{s}m,i_{s}l}\log\frac{p_{i_{s}l}P_{j_{s}m,i_{s}l}}{\sum_{l^{\prime }}\sum_{m^{\prime }}p_{i_{s}l^{\prime }}q_{j_{s}m^{\prime }}}$$

subject to the following constraints:

(6) $$\begin{array}{c} P_{D}=\left\{P_{j_{s}m,i_{s}l}:\sum_{i_{s}}\sum_{j_{s}}\sum_{l}\sum_{m}p_{i_{s}l}P_{j_{s}m,i_{s}l}{\tilde{d}}_{i_{s}j_{s}}\leq D\right\}\\ \sum_{j_{s}}\sum_{m}P_{j_{s}m,i_{s}l}=1,\;\forall i_{s}=1,2,\cdots ,\tilde{N},l=1,2,\cdots ,N_{i_{s}}\\ P_{j_{s}m,i_{s}l}\geq 0,\;\forall i_{s},m,j_{s},l \end{array}.$$

The EBA algorithm solves this optimization problem through iterative updates of the transition and reconstruction distributions. The main steps are outlined below, with a detailed pseudo-code provided in Algorithm 1.

(i) For a fixed $q_{j_{s}m}$, the transition distribution ${\overset{˘}{P}}_{j_{s}m,i_{s}l}$ is calculated with a multiplier $\lambda \in R^{+}$:

(7) $${\overset{˘}{P}}_{j_{s}m,i_{s}l}=\frac{\sum_{m^{\prime }}q_{j_{s}m^{\prime }}e^{-\lambda {\tilde{d}}_{i_{s}j_{s}}}}{\sum_{j_{s}}\sum_{m^{\prime }}q_{j_{s}m^{\prime }}e^{-\lambda {\tilde{d}}_{i_{s}j_{s}}}}.$$

(ii) For a fixed $P_{j_{s}m,i_{s}l}$, the reconstruction distribution ${\overset{˘}{q}}_{j_{s}m}$ is calculated as follows:

(8) $${\overset{˘}{q}}_{j_{s}m}=\sum_{i_{s}}\sum_{l}p_{i_{s}l}P_{j_{s}m,i_{s}l}.$$

**Algorithm 1:** EBA algorithm for the semantic RD function.
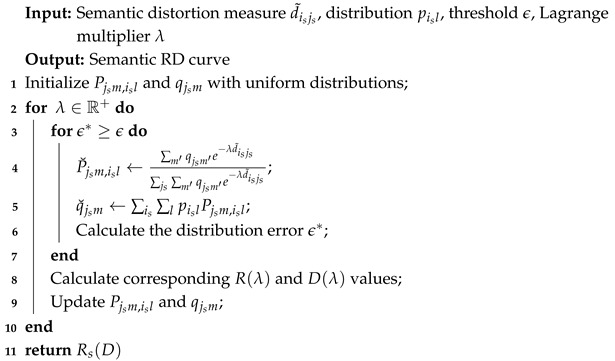


    In the following, a detailed derivation of the EBA algorithm will be provided for the semantic RD function. Similar to CIT, the optimization problem can be solved using the method of Lagrange multipliers, as the convexity of the semantic RD function has been proved in SIT [24]. For a fixed initial distribution $q_{j_{s}m}$, the problem can be formulated as finding the optimal distribution ${\overset{˘}{P}}_{j_{s}m,i_{s}l}$ that minimizes the objective function under the constraints:

(9) $$\sum_{i_{s}}\sum_{j_{s}}\sum_{l}\sum_{m}p_{i_{s}l}P_{j_{s}m,i_{s}l}{\tilde{d}}_{i_{s}j_{s}}=D$$

and

(10) $$\sum_{j_{s}}\sum_{m}P_{j_{s}m,i_{s}l}=1,\;\forall i_{s},l.$$

Beginning with a choice of Lagrange multipliers λ and $r_{i_{s}l}$ corresponding to the above constraints, the objective function is constructed as

(11) $$\begin{array}{cc} L(\lambda )= & \sum_{i_{s}}\sum_{j_{s}}\sum_{l}\sum_{m}p_{i_{s}l}P_{j_{s}m,i_{s}l}\log\frac{p_{i_{s}l}P_{j_{s}m,i_{s}l}}{\sum_{l^{\prime }}\sum_{m^{\prime }}p_{i_{s}l^{\prime }}q_{j_{s}m^{\prime }}}\\ \; & +\lambda \sum_{i_{s}}\sum_{j_{s}}\sum_{l}\sum_{m}p_{i_{s}l}P_{j_{s}m,i_{s}l}{\tilde{d}}_{i_{s}j_{s}}\\ \; & +\sum_{i_{s}}\sum_{l}r_{i_{s}l}\sum_{j_{s}}\sum_{m}P_{j_{s}m,i_{s}l} \end{array}.$$

To find the optimal solution, the partial derivative of L(λ) with respect to $P_{j_{s1}m_{1},i_{s1}l_{1}}$ is taken and set to zero,

(12) $$\begin{array}{cc} \frac{\partial L(\lambda )}{\partial P_{j_{s1}m_{1},i_{s1}l_{1}}} & =p_{i_{s1}l_{1}}\left[\log\frac{p_{i_{s1}l_{1}}P_{j_{s1}m_{1},i_{s1}l_{1}}}{\sum_{l^{\prime }}p_{i_{s1}l^{\prime }}}+1\right]-p_{i_{s1}l_{1}}\log\sum_{m^{\prime }}q_{j_{s1}m^{\prime }}+\lambda p_{i_{s1}l_{1}}{\tilde{d}}_{i_{s1}j_{s1}}+r_{i_{s1}l_{1}}\\ \; & =p_{i_{s1}l_{1}}\log\frac{p_{i_{s1}l_{1}}P_{j_{s1}m_{1},i_{s1}l_{1}}}{\sum_{l^{\prime }}p_{i_{s1}l^{\prime }}\sum_{m^{\prime }}q_{j_{s1}m^{\prime }}}+p_{i_{s1}l_{1}}+\lambda p_{i_{s1}l_{1}}{\tilde{d}}_{i_{s1}j_{s1}}+r_{i_{s1}l_{1}}=0 \end{array}.$$

Solving for $P_{j_{s1}m_{1},i_{s1}l_{1}}$, thus obtaining

(13) $$P_{j_{s1}m_{1},i_{s1}l_{1}}=p_{i_{s1}l_{1}}^{-1}\sum_{l^{\prime }}p_{i_{s1}l^{\prime }}\sum_{m^{\prime }}q_{j_{s1}m^{\prime }}e^{-\lambda {\tilde{d}}_{i_{s1}j_{s1}}-p_{i_{s1}l_{1}}^{-1}r_{i_{s1}l_{1}}-1}.$$

By substituting $\left(i_{s1},j_{s1},l_{1},m_{1}\right)\to \left(i_{s},j_{s},l,m\right)$ and applying the normalization constraint, we derive

(14) $${\overset{˘}{P}}_{j_{s}m,i_{s}l}=\frac{p_{i_{s}l}^{-1}\sum_{l^{\prime }}p_{i_{s}l^{\prime }}\sum_{m^{\prime }}q_{j_{s}m^{\prime }}e^{-\lambda {\tilde{d}}_{i_{s}j_{s}}-p_{i_{s}l}^{-1}r_{i_{s}l}-1}}{\sum_{j_{s}}\sum_{m}p_{i_{s}l}^{-1}\sum_{l^{\prime }}p_{i_{s}l^{\prime }}\sum_{m^{\prime }}q_{j_{s}m^{\prime }}e^{-\lambda {\tilde{d}}_{i_{s}j_{s}}-p_{i_{s}l}^{-1}r_{i_{s}l}-1}}.$$

The distribution ${\overset{˘}{P}}_{j_{s}m,i_{s}l}$ is calculated for the fixed $q_{j_{s}m}$, as shown in Equation (7). Subsequently, with the transition probability distribution ${\overset{˘}{P}}_{j_{s}m,i_{s}l}$ fixed, the reconstruction distribution ${\overset{˘}{q}}_{j_{s}m}$ is calculated according to Equation (8). Based on these updated distributions, the distribution error

(15) $$\epsilon^{*}=\sum_{i_{s},l,j_{s},m}\left|{\overset{˘}{P}}_{j_{s}m,i_{s}l}-P_{j_{s}m,i_{s}l}\right|+\sum_{j_{s},m}\left|{\overset{˘}{q}}_{j_{s}m}-q_{j_{s}m}\right|$$

is calculated. If $\epsilon^{*}$ is less than the predetermined threshold ϵ, the EBA algorithm is considered to have converged for the current λ, and the corresponding RD pair (R(λ),D(λ)) is obtained by

(16) $$\begin{array}{c} R\left(\lambda \right)=\sum_{i_{s}}\sum_{j_{s}}\sum_{l}\sum_{m}p_{i_{s}l}{\overset{˘}{P}}_{j_{s}m,i_{s}l}\log\frac{p_{i_{s}l}{\overset{˘}{P}}_{j_{s}m,i_{s}l}}{\sum_{l^{\prime \prime }}\sum_{m^{\prime \prime }}p_{i_{s}l^{\prime \prime }}{\overset{˘}{q}}_{j_{s}m^{\prime \prime }}}\\ D\left(\lambda \right)=\sum_{i_{s}}\sum_{j_{s}}\sum_{l}\sum_{m}p_{i_{s}l}{\overset{˘}{P}}_{j_{s}m,i_{s}l}{\tilde{d}}_{i_{s}j_{s}} \end{array}.$$

Otherwise, these distributions are updated as the initial distributions for the next iteration, i.e.,

(17) $$\begin{array}{cc} P_{j_{s}m,i_{s}l} & ={\overset{˘}{P}}_{j_{s}m,i_{s}l}\\ q_{j_{s}m} & ={\overset{˘}{q}}_{j_{s}m} \end{array}.$$

The entire semantic RD function curve can be obtained by sweeping through the value of λ, where each (R(λ),D(λ)) pair corresponds to a point on the curve. The EBA algorithm incorporates synonymous mappings, enabling it to compute the semantic rate-distortion function, while the classical BA algorithm operates solely at the syntactic symbol level and does not account for any semantic structure or synonymous relationships.

### 3.2. EBA Algorithm for the Optimal Synonymous Mapping

When the synonymous mapping is unknown, an optimization framework that combines the EBA algorithm with simulated annealing is introduced to find the optimal synonymous mapping (i.e., the mapping with maximum synonymous number). To formally characterize the synonymous number, we introduce the following definition.

**Definition** **1.** *The synonymous number $L_{s}$ is defined by*

(18) $$L_{s}=\sum_{i_{s}}p_{i_{s}}N_{i_{s}}$$

*where $N_{i_{s}}$ represents the number of syntactic symbols mapped to the $i_{s}$-th semantic symbol.*

As mentioned in [24], it is worth noting that the semantic RD function is not necessarily non-negative, thus requiring specific objective constraints to ensure meaningful results. In this paper, the distortion value at $R_{s}=0$ is considered as the constraint, which represents the maximum achievable distortion when no information is transmitted. For convenience, we denote the synonymous mapping as *M*. Algorithm 2 describes the optimization process.
**Algorithm 2:** Optimization framework for finding the optimal synonymous mapping.
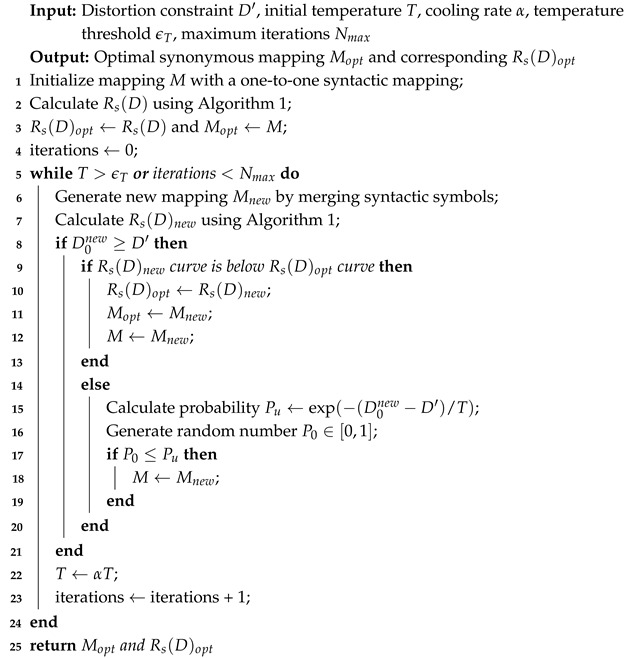


The inputs to the optimization framework include the distortion constraint $D^{\prime }$, the initial temperature *T*, the cooling rate α, the temperature threshold $\epsilon_{T}$, and the maximum number of iterations $N_{max}$. The outputs are the optimal synonymous mapping $M_{opt}$ (which achieves the maximum $L_{s}$ under the constraint $D=D^{\prime }$ when $R_{s}\left(D\right)=0$) and the corresponding semantic RD function $R_{s}{\left(D\right)}_{opt}$.

In the proposed optimization framework, a syntactic mapping is considered as the initial synonymous mapping *M*, where one semantic symbol corresponds to one syntactic symbol. Given a source distribution $p_{i_{s}l}$ and a distortion measure ${\tilde{d}}_{i_{s}j_{s}}$, the problem is transformed into calculating the semantic RD function under a given synonymous mapping, which can be directly solved using the EBA algorithm as shown in Algorithm 1. Then set $M_{opt}=M$, $R_{s}{\left(D\right)}_{opt}=R_{s}\left(D\right)$.

Initialize the iteration number to zero. While the current temperature *T* is larger than the threshold $\epsilon_{T}$ or the number of iterations is less than the maximum limit $N_{max}$, the following steps are executed:

At each iteration, based on the current mapping and the probability distribution, which determines the probability of merging adjacent syntactic symbols into one semantic symbol, a new semantic mapping $M_{new}$ is generated. The new semantic RD curve $R_{s}{\left(D\right)}_{new}$ is then computed using the EBA algorithm. The corresponding $D_{0}^{new}$ is defined as the value of *D* when $R_{s}\left(D\right)new=0$. If $D_{0}^{new}$ is less than the constraint $D^{\prime }$, indicating that $M_{new}$ does not meet the optimization objective and thus needs to be regenerated. Otherwise, it is checked whether the $R_{s}{\left(D\right)}_{new}$ curve lies below the current $R_{s}\left(D\right)$ curve. If so, both the optimal mapping $M_{opt}$ and current mapping *M* are updated. To avoid the local optimum solutions, even if $M_{new}$ yields a higher semantic RD curve, it may still be accepted with probability

(19) $$P_{u}=e^{-\frac{D_{0}^{new}-D^{\prime }}{T}}.$$

The temperature *T* and the number of iterations are updated after each iteration.

The algorithm terminates when $T\leq \epsilon_{T}$ or the iterations reach $N_{max}$, returning the optimal mapping $M_{opt}$ and its corresponding semantic RD function $R_{s}{\left(D\right)}_{opt}$. Through the combination of the EBA algorithm and simulated annealing, the optimal synonymous mapping $M_{opt}$ under the given constraints can be found.

## 4. The Application to SKB

SKB is a key concept in the semantic communication system that facilitates the semantic encoding and decoding process between the transmitter and the receiver [28,29,30,31]. For each mobile intelligent agent, it defines a set of semantic knowledge vectors corresponding to different classes, which can be obtained from Word2Vec or manually annotated semantic attribute vectors [32]. These vectors not only characterize the attribute features of corresponding classes but also reflect the semantic relationships among different classes. Specifically, we denote $W≜\{w_{1},w_{2},\cdots ,w_{N}\}$ as the set of syntactic vectors and $S≜\{s_{1},s_{2},\cdots ,s_{K}\}$ as the set of semantic vectors, where semantic vector $s_{i}$ is referred to as the semantic prototype of the *i*-th class. It is worth noting that both transmitter and the receiver use the same SKB to ensure the effectiveness of semantic communication.

From the perspective of SIT, the SKB can be considered as a specific instance of synonymous mapping, where each semantic vector *s* corresponds to a semantic symbol $\tilde{x}$ defined in Section 2, and syntactic vectors *w* sharing similar semantic vectors can be regarded as syntactic symbols *x* mapped to the same semantic symbol $\tilde{x}$. Therefore, for a given source, the semantic RD function can be directly computed using the EBA algorithm by considering the SKB as a synonymous mapping. By varying the SKB size, different semantic RD function results can be obtained. Through analyzing the relationship between the semantic RD function and the SKB size, the semantic communication compression efficiency can be quantitatively evaluated, which has theoretical significance for guiding the design of the semantic communication system.

**Figure 2:**
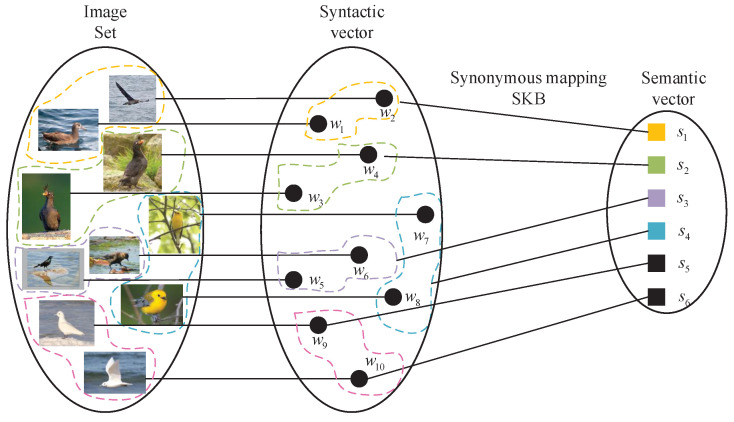
Illustration of the SKB as a synonymous mapping. The left part shows the syntactic samples, the middle part shows the set of syntactic vectors W, and the right part shows the set of semantic vectors S.

An example is presented in Figure 2 to illustrate the SKB as a synonymous mapping. The left part shows the syntactic samples, the middle part presents the set of syntactic vectors W, and the right part displays the set of semantic vectors S. For this example, a dataset containing 10 uniformly distributed samples from 5 different bird classes is considered, with 2 samples per class (samples of the same class are circled by dashed lines). These images are first converted into vector representations to obtain their corresponding syntactic vectors. Obviously, the probability distribution is given by p(w)={0.1,0.1,⋯,0.1}. Assuming that the SKB contains semantic attributes of 4 bird classes, these syntactic vectors can be mapped to the corresponding semantic vector space. Specifically, this mapping can be expressed as: $\left\{w_{1},w_{2}\right\}\to s_{1}$, $\left\{w_{3},w_{4}\right\}\to s_{2}$, $\left\{w_{5},w_{6}\right\}\to s_{3}$, $\left\{w_{7},w_{8}\right\}\to s_{4}$. However, for the remaining bird class whose semantic attributes are not included in the SKB, its two syntactic vectors correspond to distinct semantic vectors in semantic space, i.e., $w_{9}\to s_{5}$ and $w_{10}\to s_{6}$. Consequently, the probability distribution p(s)={0.2,0.2,0.2,0.2,0.1,0.1} is obtained. Based on p(w) and p(s), the semantic RD result can be computed using the EBA algorithm.

## 5. Experimental Results

In this section, the simulation results of the semantic RD function calculated by the EBA algorithm are presented. The semantic mean squared error (SMSE) distortion is adopted, which is defined as

(20) $${\tilde{d}}_{i_{s}j_{s}}=\left\{\begin{array}{cc} 0, & \;i_{s}=j_{s}\\ \frac{1}{l\cdot m}\cdot \sum_{l}\sum_{m}{∥x_{i_{s},l}-{\hat{x}}_{j_{s},m}∥}^{2}, & i_{s}\neq j_{s} \end{array}\right..$$

**Figure 3:**
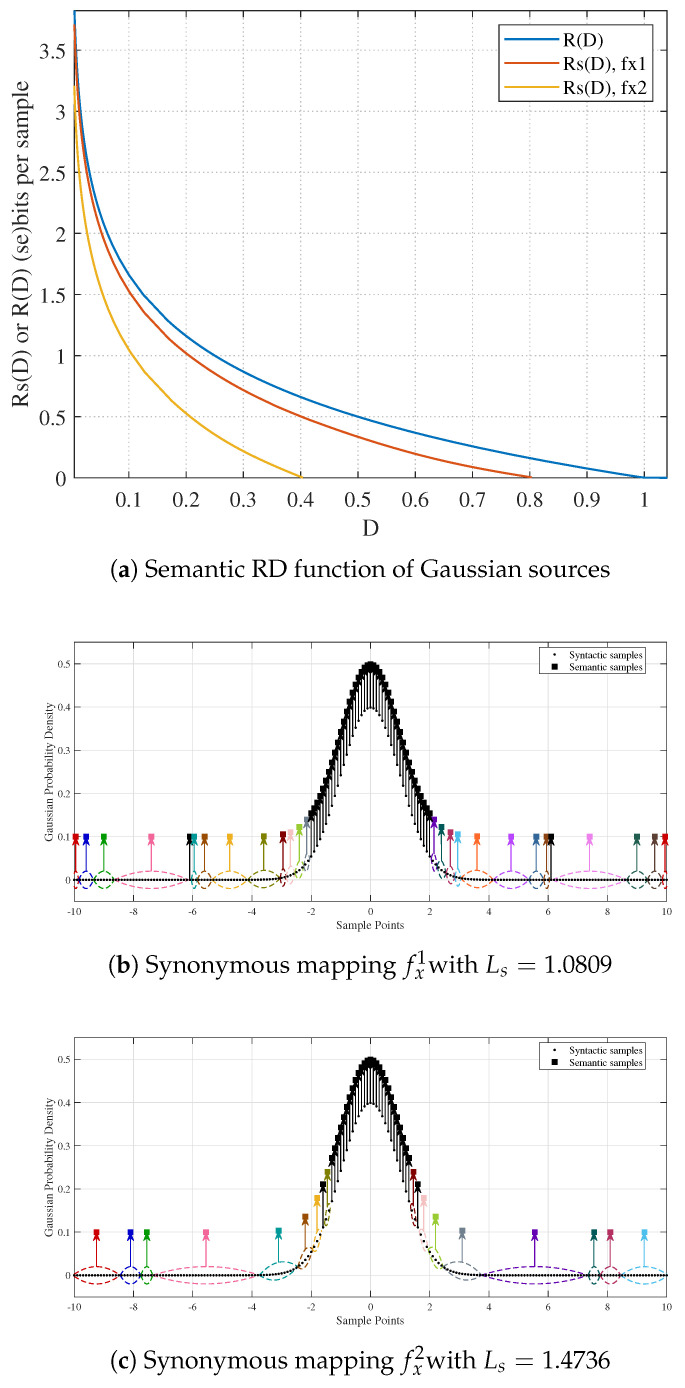
Semantic RD function of Gaussian sources under the given synonymous mappings.

Figure 3a shows the semantic RD function of Gaussian sources with zero mean and variance $\sigma^{2}=1$ computed by the EBA algorithm under the given synonymous mappings $f_{x}$, where the blue curve represents the syntactic RD function of a Gaussian source. For the Gaussian source, 201 syntactic points are uniformly sampled in the interval [−10,10], and their corresponding probability distribution p(x) follows the Gaussian distribution. Based on $f_{x}$, $p(\tilde{x})$ can be easily obtained. Figure 3b,c illustrate two synonymous mappings $f_{x}^{1}$ and $f_{x}^{2}$ with $L_{s}=1.0809$ and $L_{s}=1.4736$, respectively. With the error threshold ϵ=0.0001, the corresponding semantic RD function can be computed using the EBA algorithm. The simulation results show that the semantic RD function decreases as the synonymous number $L_{s}$ grows and becomes significantly lower than the classic counterparts. Furthermore, it can be observed that when $L_{s}=1$, the semantic RD function is identical to the classic RD function.

**Figure 4:**
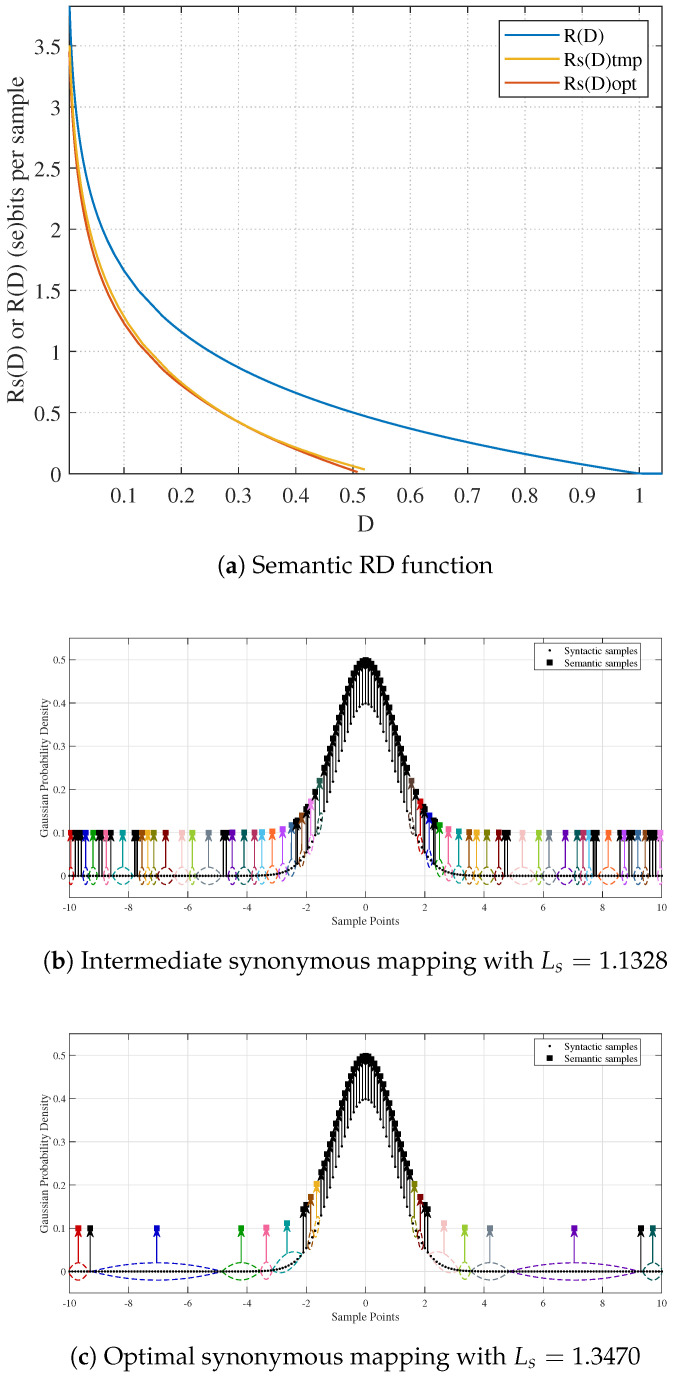
Semantic RD function and optimal synonymous mapping obtained by the optimization algorithm.

Given the distortion constraint $D^{\prime }$, the optimal synonymous mapping can be obtained using Algorithm 2. The simulated annealing parameters are set as follows: initial temperature T=100, maximum iteration number $N_{max}=1000$, cooling rate α=0.99, and temperature threshold $\epsilon_{T}=10^{-6}$. For the aforementioned Gaussian source, 201 syntactic points are uniformly sampled in the interval [−10,10]. The interval merging is performed as follows: based on the probability distribution (a mixture of two Gaussian distributions centered at −10 and 10), an interval endpoint is first located and extended by δ, where δ ranges from −0.5 to 0.5 with a step size of 0.1. Then, multiple syntactic points within this extended range are merged into a semantic representation. Finally, to maintain symmetry, the same merging process is applied to the corresponding symmetric interval. With $D^{\prime }=0.5$, the optimization results are presented in Figure 4. Specifically, Figure 4b shows an intermediate synonymous mapping with $L_{s}=1.1328$ during the optimization process, while Figure 4c presents the final optimal synonymous mapping with $L_{s}=1.3470$. The results clearly show that the optimization algorithm finds the synonymous mapping with the maximum $L_{s}$ under the given distortion constraint. Figure 4a illustrates the corresponding semantic RD function, where the blue curve represents the syntactic RD function of a Gaussian source. It should be noted that the optimization problem may yield multiple optimal synonymous mappings under the current constraint setting, and additional constraints would be necessary to ensure uniqueness.

**Figure 5:**
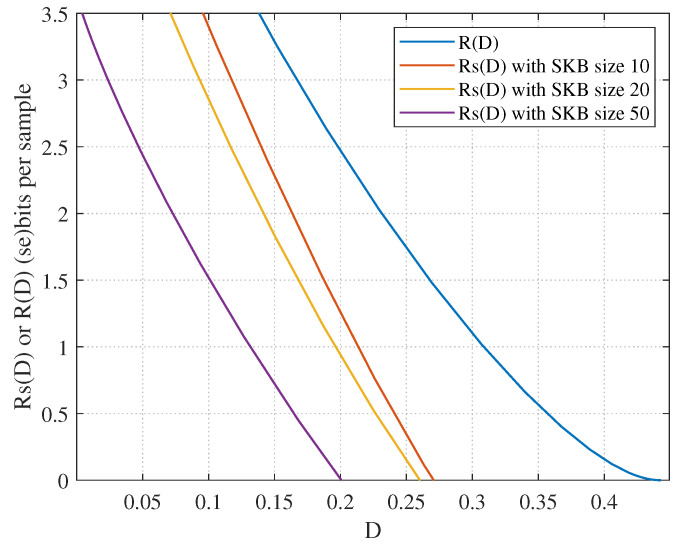
Impact of SKB size on the semantic RD function.

Using the CUB dataset [27], the test set can be seen as an SKB containing 50 feature vectors; 2 samples are selected from each class, resulting in a total of 100 test samples. To investigate the impact of SKB size on the semantic RD function, the number of test samples is fixed and three SKB configurations are considered: 10 classes, 20 classes, and the complete 50 classes from the CUB test set. Figure 5 shows the semantic RD results under different SKB sizes, where the blue curve represents the syntactic RD function for transmitting 100 samples without the assistance of SKB. The simulation result shows that as the SKB size increases, the semantic RD function decreases significantly, indicating improved compression efficiency. Furthermore, compared with the syntactic RD function, the simulation result demonstrates the effectiveness of the SKB in improving compression performance. In practical applications, the semantic RD function can be computed by the EBA algorithm and then utilized for theoretical analysis to predict the required SKB size and compression limits based on specific task objectives.

## 6. Conclusions

In this paper, we extend the classic BA algorithm to semantic communication based on synonymous mapping and propose the EBA algorithm, which can compute the semantic RD function for a given source. Furthermore, by combining it with the optimization algorithm, the optimal synonymous mapping can be obtained under given constraints. From a practical perspective, by considering the SKB as a given synonymous mapping, the EBA algorithm provides an approach for analyzing the SKB size, thereby offering theoretical insights into the trade-off between semantic distortion and compression performance. Future work could explore the application of the EBA algorithm in other scenarios, establishing a more comprehensive theoretical foundation for the semantic communication system.

## Data Availability

The original contributions presented in this study are included in the article. Further inquiries can be directed to the corresponding author.

## Abbreviations

The following abbreviations are used in this manuscript:

BA	Blahut–Arimoto
CIT	classical information theory
CUB	CUB-200-2011 Birds
EBA	extended Blahut–Arimoto
RD	rate-distortion
SIT	semantic information theory
SKB	semantic knowledge base
SMSE	semantic mean squared error

## References

[1] Gündüz D., Qin Z., Aguerri I.E., Dhillon H.S., Yang Z., Yener A., Wong K.K., Chae C.B. (2022). Beyond transmitting bits: Context, semantics, and task-oriented communications. IEEE J. Sel. Areas Commun..

[2] Weaver W. (2017). The mathematics of communication. Communication Theory.

[3] Farsad N., Rao M., Goldsmith A. Deep learning for joint source-channel coding of text. Proceedings of the 2018 IEEE International Conference on Acoustics, Speech and Signal Processing (ICASSP).

[4] Xie H., Qin Z., Li G.Y., Juang B.H. (2021). Deep learning enabled semantic communication systems. IEEE Trans. Signal Process..

[5] Bourtsoulatze E., Kurka D.B., Gündüz D. (2019). Deep joint source-channel coding for wireless image transmission. IEEE Trans. Cogn. Commun. Netw..

[6] Weng Z., Qin Z. (2021). Semantic communication systems for speech transmission. IEEE J. Sel. Areas Commun..

[7] Shannon C.E. (1948). A mathematical theory of communication. Bell Syst. Tech. J..

[8] Shannon C.E. (1993). Coding Theorems for a Discrete Source With a Fidelity CriterionInstitute of Radio Engineers, International Convention Record, vol. 7, 1959. Claude E. Shannon: Collected Papers.

[9] Berger T. (2003). Rate-distortion theory. Wiley Encyclopedia of Telecommunications.

[10] Cover T.M. (1999). Elements of Information Theory.

[11] Arimoto S. (1972). An algorithm for computing the capacity of arbitrary discrete memoryless channels. IEEE Trans. Inf. Theory.

[12] Blahut R. (1972). Computation of channel capacity and rate-distortion functions. IEEE Trans. Inf. Theory.

[13] Dupuis F., Yu W., Willems F.M. Blahut-Arimoto algorithms for computing channel capacity and rate-distortion with side information. Proceedings of the International Symposium onInformation Theory, ISIT 2004.

[14] Lingyi C., Wu S., Ye W., Wu H., Zhang W., Wu H., Bo B. (2025). A Constrained BA Algorithm for Rate-Distortion and Distortion-Rate Functions. Csiam Trans. Appl. Math..

[15] Matz G., Duhamel P. Information geometric formulation and interpretation of accelerated Blahut-Arimoto-type algorithms. Proceedings of the Information Theory Workshop.

[16] Sayir J. Iterating the Arimoto-Blahut algorithm for faster convergence. Proceedings of the 2000 IEEE International Symposium on Information Theory (Cat. No. 00CH37060).

[17] Yu Y. (2010). Squeezing the Arimoto–Blahut algorithm for faster convergence. IEEE Trans. Inf. Theory.

[18] Rose K. (1994). A mapping approach to rate-distortion computation and analysis. IEEE Trans. Inf. Theory.

[19] Stavrou P.A., Kountouris M. (2023). The role of fidelity in goal-oriented semantic communication: A rate distortion approach. IEEE Trans. Commun..

[20] Serra G., Stavrou P.A., Kountouris M. (2024). Alternating Minimization Schemes for Computing Rate-Distortion-Perception Functions with *f*-Divergence Perception Constraints. arXiv.

[21] Serra G., Stavrou P.A., Kountouris M. Computation of rate-distortion-perception function under f-divergence perception constraints. Proceedings of the 2023 IEEE International Symposium on Information Theory (ISIT).

[22] Li D., Huang J., Huang C., Qin X., Zhang H., Zhang P. (2023). Fundamental limitation of semantic communications: Neural estimation for rate-distortion. J. Commun. Inf. Netw..

[23] Liang Z., Niu K., Wang C., Xu J., Zhang P. (2025). Synonymous Variational Inference for Perceptual Image Compression. arXiv.

[24] Niu K., Zhang P. (2024). A mathematical theory of semantic communication. J. Commun..

[25] Farquhar S., Kossen J., Kuhn L., Gal Y. (2024). Detecting hallucinations in large language models using semantic entropy. Nature.

[26] Rose K. (1998). Deterministic annealing for clustering, compression, classification, regression, and related optimization problems. Proc. IEEE.

[27] Wah C., Branson S., Welinder P., Perona P., Belongie S. (2011). The Caltech-UCSD Birds-200–2011 Dataset.

[28] Ren J., Zhang Z., Xu J., Chen G., Sun Y., Zhang P., Cui S. (2024). Knowledge base enabled semantic communication: A generative perspective. IEEE Wirel. Commun..

[29] Ni F., Wang B., Li R., Zhao Z., Zhang H. (2025). Interplay of semantic communication and knowledge learning. Wireless Semantic Communications: Concepts, Principles and Challenges.

[30] Hello N., Di Lorenzo P., Strinati E.C. Semantic communication enhanced by knowledge graph representation learning. Proceedings of the 2024 IEEE 25th International Workshop on Signal Processing Advances in Wireless Communications (SPAWC).

[31] Yi P., Cao Y., Kang X., Liang Y.C. (2023). Deep learning-empowered semantic communication systems with a shared knowledge base. IEEE Trans. Wirel. Commun..

[32] Sun Y., Chen H., Xu X., Zhang P., Cui S. (2023). Semantic knowledge base-enabled zero-shot multi-level feature transmission optimization. IEEE Trans. Wirel. Commun..

